# Risk Factors in Post-LASIK Corneal Ectasia

**DOI:** 10.1155/2014/204191

**Published:** 2014-06-03

**Authors:** Mehmet Gurkan Tatar, Feride Aylin Kantarci, Aydin Yildirim, Haşim Uslu, Hatice Nur Colak, Hasan Goker, Bulent Gurler

**Affiliations:** ^1^Department of Ophthalmology, Medicine Faculty, Fatih University, Eski Londra Asfaltı, Hürriyet Mah. Dumlupınar Sokak No. 10, Şirinevler-Bahçelievler, 34192 İstanbul, Turkey; ^2^Department of Ophthalmology, Medicine Faculty, Fatih University, Yalı Mah. Sahil Yolu Sokak No. 16, Maltepe, 34844 İstanbul, Turkey; ^3^Department of Ophthalmology, Medicine Faculty, Fatih University, Atatürk M. Sütçü İmam Caddesi No. 14, Ümraniye, 34764 İstanbul, Turkey

## Abstract

*Purpose*. To evaluate the risk factors for post-laser in situ keratomileusis (LASIK) ectasia. *Materials and Methods*. Medical records of 42 eyes of 28 (10 women, 18 men) patients who developed corneal ectasia after LASIK were retrospectively reviewed. Topographical features and surgical parameters of those patients were evaluated. *Results*. The mean age of patients was 34.73 ± 6.50 (23–48) years and the mean interval from LASIK to the diagnosis of post-LASIK ectasia was 36.0 ± 16.92 (12–60) months. The following factors were determined as a risk factors: deep ablation (>75 **μ**m) in 10 eyes, FFK (forme fruste keratoconus) in 6 eyes, steep cornea (>47 D) in 3 eyes, pellucid marginal degeneration (PMD) in 2 eyes, thin cornea (<500 **μ**m) in 2 eyes, thin and steep cornea in 2 eyes, thin cornea and deep ablation in 5 eyes, FFK and steep cornea in 2 eyes, and FFK, steep cornea, and deep ablation in 1 eye. However no risk factor has been determined in 9 eyes (21.4%). *Conclusion*. The findings of our study showed that most of the patients who developed post-LASIK ectasia have a risk factor for post-LASIK ectasia. However, the most common risk factor was deep ablation.

## 1. Introduction

Post-laser in situ keratomileusis (LASIK) corneal ectasia is one of the most feared complications of refractive surgery. Post-LASIK ectasia was described first by Seiler et al. [1] as progressive stromal thinning, corneal steepening, decreased uncorrected distance visual acuity (UDVA), and corrected distance visual acuity (CDVA). Although exact prevalence of post-LASIK ectasia is not known, reported prevalence rates change between 0.02% and 0.6% [2–4].

Corneal thickness lower than 500 *μ*m, residual stromal bed thickness lower than 250 *μ*m, deep ablation, and abnormal corneal topography are among preoperative risk factors for corneal ectasia [5–7]. Randleman et al. [8] developed a scale, ectasia risk score system (ERSS), which includes preoperative parameters, to rate post-LASIK ectasia. This scale includes risk factors such as low stromal bed thickness, low preoperative corneal thickness, abnormal preoperative corneal topography,   young age, and high refractive correction. Therefore, it is very important to determine preoperative risk factors before LASIK and to select cases regarding these risk factors to prevent corneal ectasia. The aim of this study is to evaluate risk factors in subjects with post-LASIK ectasia.

## 2. Materials and Methods

This study included subjects with post-LASIK ectasia from Private Turkey Hospital Eye Clinic and Departments of Ophthalmology, Medical Faculty, Fatih University, between 1999 and 2010. Data were evaluated retrospectively. Ethical board review was obtained and study was conducted according to Declaration of Helsinki.

### 2.1. Preoperative Evaluation

Along with detailed ophthalmological examinations, age, gender, and time of corneal ectasia diagnosis were recorded. UDVA and CDVA values per Snellen chart, spherical and astigmatic refraction, corneal topography (Orbscan II, Bausch & Lomb, Orbtek, Inc.,) findings, and central corneal thickness (CCT) measurement with ultrasonic pachymeter (TOMEY AL-3000, Tomey Corp., Nagoya, Japan.) were evaluated.

### 2.2. Intraoperative Data

Patients were operated on by using the VISX S4 IR excimer laser system (Abbott Medical Optics). The flaps were created with the Moria M2 single use 130 *μ*m microkeratome head (Moria, Antony, France).

### 2.3. Patient Selection

Clinical diagnosis of corneal ectasia was made by progressive central or inferior corneal steepening, increased myopia and/or astigmatism, and decreased UDVA and CDVA [7]. Subjects who had glaucoma diagnosis, other corneal surgeries (other than LASIK), and central corneal opacity were excluded.

### 2.4. Description of Risk Factors

Thin cornea was described as preoperative CCT less than 500 *μ*m; steep cornea was described as keratometric value higher than 47 D; deep ablation was described as corneal tissue ablation more than 75 *μ*m; and forme fruste keratoconus (FFK) was described as 1.4 D or higher difference between inferior and superior keratometric values at corneal topography.

### 2.5. Data Analysis

SPSS 16 (statistical package for social sciences) software was used for data entry and statistical analysis. Student's *t*-test and chi-square test were used in the analysis. Statistical significance was defined as *P* < 0.05.

## 3. Results

### 3.1. Demographical Data

Forty-four eyes of 30 patients (10 women, 20 men) who were followed up for post-LASIK corneal ectasia were evaluated retrospectively. Two eyes of 2 patients were excluded since pre-LASIK topographical data were lacking. 42 eyes of 28 patients in the study were evaluated. The mean age of patients was 34 ± 6.5 (23–48) years and the mean time of diagnosis of corneal ectasia was 36 ± 16.92 (12–60) months. Demographical features of the patients are summarized in Table 1.

### 3.2. Clinical and Topographic Data

The preoperative mean UDVA was 0.18 ± 0.22, preoperative mean CDVA was 0.46 ± 0.26, preoperative mean spherical value was −3.40 ± 4.29, mean preoperative keratometric value was 45.69 ± 4.63 D, preoperative mean cylindrical value was −3.10 ± 1.59D , and mean CCT was 447.43 ± 47.17 *μ*m. Preoperative findings including UDVA, CDVA, CCT, *K*_1_, *K*_2_, *K*_mean_, spherical value, cylindrical value, and time of corneal ectasia diagnosis after LASIK were summarized in Table 2.

Risk factors were detected as deep ablation in 10 eyes, FFK in 6 eyes, steep cornea in 3 eyes, PMD in 2 eyes, thin cornea in 2 eyes, thin and steep cornea in 2 eyes, thin cornea and deep ablation in 5 eyes, and FFK and steep cornea in 2 eyes. While there was at least one risk factor in 33 (78.5%) eyes with post-LASIK ectasia, there were no risk factors in 9 (21.4%) eyes. Distribution of preoperative risk factors in the patients was summarized in Table 3.

## 4. Discussion

Post-LASIK corneal ectasia is a rare but a serious complication which leads to decreased visual acuity. Treatment options are available with keratoplasty as the last resort. Ectasia prevalence can be decreased by determining risk factors which lead to post-LASIK corneal ectasia. Refractive surgery decreases collagen tension, by disrupting cornea biomechanics, and may lead to corneal ectasia [2]. Stromal thinning, anterior and posterior corneal steepening, progressive increase in myopia, irregular astigmatism, and decreased UDVA and CDVA may be seen after corneal ectasia [7, 9]. Mean time of corneal ectasia after LASIK is reported as 13 months (6–20 months) [10].

Subjects with FFK in corneal topography are at the highest risk for ectasia [2, 11, 12]. Since incidence of FFK is higher among cases with refractive surgery when compared with general population and since the refractive surgery increases the progress of the disorder, pre-LASIK evaluation is very important [1, 2, 13]. Brenner et al. [14] found preoperative FFK in 58 of 77 (75.3%) patients with post-LASIK ectasia. On the other hand, Leccisotti [15] reported ectasia incidence after PRK (photorefractive keratectomy) as 0.03%. Preoperative FFK was found in 3 of 5 subjects with ectasia in the same study [15]. In our study, we detected FFK in 9 (21.4%) patients as a risk factor. On the other hand, Jampaulo and Maloney [16] did not report ectasia in a patient they followed up for 7 years after LASIK with preoperative keratoconus (right eye FFK and left eye inferior corneal steepening).

In some articles, subjects with PMD (pellucid marginal degeneration) are reported to be at higher risk for post-LASIK corneal ectasia [17–19]. Ambrósio and Wilson [18] showed that corneal ectasia developed after refractive surgery in 2 subjects with early PMD. In another study, preoperative PMD was detected in 3 eyes with post-LASIK ectasia [17]. In our study, we found PMD as a risk factor in 2 (4.8%) patients.

Preoperative abnormal topographical findings have an important place in development of post-LASIK corneal ectasia. Randleman [11] found risk of corneal ectasia higher in patients with preoperative asymmetrical inferior corneal steepening, asymmetric bowtie patterns, and eyes with superior or inferior skewed steepening when compared with normal population. Randleman et al. [20] found abnormal preoperative topographical findings in 50% of patients with ectasia. Spadea et al. [21] detected preoperative abnormal topographical findings in 8 of 23 (34.8%) subjects with post-LASIK ectasia. On the contrary, Said et al. [22] reported normal preoperative topographical findings in 29 eyes with ectasia. In our study, we detected abnormal preoperative topographical findings in 11 of 42 eyes (26.2%). Randleman et al. [7] reported that preoperative cornea steeper than 47 D is a risk factor for ectasia. In our study we found steep cornea in 8 (19.04%) subjects.

One of the reasons of corneal ectasia is weakened residual stromal bed due to loss of large amount of stromal tissue because of high myopia correction. This weakening may also be due to producing a thicker flap than planned [23]. In a study comparing subjects with or without post-LASIK ectasia, higher myopia (−8 D myopia) was detected in those with ectasia [7]. Pallikaris et al. [24] showed that ectasia develops in different residual stromal thicknesses in patients with myopia higher than 10 D. In our study, we found myopia higher than −8 D in 8 eyes (19.04%).

Some studies reported that in subjects with normal corneal topographical findings and residual stromal beds, CCT lower than 500 *μ*m is not a risk factor for ectasia [2, 25, 26]. There is no consensus on reliable residual stromal bed thickness for post-LASIK corneal ectasia development. Barraquer [27] does not suggest LASIK application in myopic eyes with CCT thinner than 450 *μ*m and/or residual stromal bed thickness less than 150 *μ*m. Brenner et al. [9] found mean CCT in eyes with post-LASIK ectasia as 534.18 ± 28.58 *μ*m. Another study reported that thin CCT is not an isolated risk factor for post-LASIK ectasia in eyes with normal topographical findings [2]. In our study, mean CCT was 447.43 ± 47.17 *μ*m (350–527) and mean time of post-LASIK ectasia was 36 ± 16.92 (12–60) months and there was deep ablation in 16 eyes (38.1%). In this study, we found thinner mean CCT than most studies.

Condon et al. [23] found preoperative mean spherical value as −15.58 ± 5.63 D (−10.00–35.00) and mean cylindrical value as −1.64 ± 5.63 D in eyes with post-LASIK ectasia; Brenner et al. [14] found preoperative CDVA as 0.86 ± 0.46, preoperative spherical value as −5.12 ± 3.90, preoperative mean cylindrical value as 1.65 ± 1.21 D, and mean preoperative keratometric value as 44.09 ± 2.26 D. Twa et al. [10] showed that preoperative CDVA is lower, CCT is thinner, and stromal ablation is higher in subjects with post-LASIK corneal ectasia when compared with subjects without ectasia. Spadea et al. [21] reported preoperative CDVA as 0.7 ± 0.27, preoperative spherical value as 8.11 ± 4.48, and preoperative keratometric value as 42.38 ± 2.06 D in subjects with LASIK application. In our study, preoperative mean UDVA was 0.18 ± 0.22 and preoperative mean CDVA was 0.46 ± 0.26; preoperative spherical value was −3.40 ± 4.29, mean preoperative keratometric value was 45.69 ± 4.63 D, and preoperative mean cylindrical value was −3.10 ± 1.59 D.

Interestingly, age has been reported as a potential risk factor and it has been suggested that subjects in this group may have emerging keratoconus or FFK [28]^.^ Although Randleman et al. [8] reported young age as a risk factor in ERSS (ectasia risk score system), Binder and Trattler [29] did not find any corneal ectasia in 150 eyes in subjects 21–29 years of age. Spadea et al. [21] reported that 4 of 23 subjects with post-LASIK corneal ectasia were young (<30 years). Keratoconus usually emerges in the second decade and it is rare after 4th decade [21]. In our study, mean age was 34 ± 6.5 years and 10 subjects (33%) were younger than 30 years of age.

Saad and Gatinel [30] reported ectasia in a case after 2 years who had no risk factor (young age, high myopia, low residual stromal bed, and CCT) and 0 ERSS in both eyes. Wang et al. [31] reported bilateral corneal ectasia in a subject with LASIK application to one eye and no surgical treatment to the other eye and who had normal topographic findings. In our study, corneal ectasia was determined in 9 patients without any risk factors.

Weakness of this paper is the retrospective nature and limited number of subjects with ectasia. However, since post-LASIK ectasia subjects have decreased considerably with the advance in LASIK surgery techniques and better subject selection by using preoperative risk analysis, it seems difficult to conduct prospective studies with large samples.

In summary, the most common risk factor for post-LASIK corneal ectasia was deep ablation in our study. FFK, thin cornea, steep cornea, and PMD follow in order of frequency. We did not find any risk factors in 9 patients. Therefore, it is important to detect preoperative risk factors in LASIK candidates to prevent ectasia.

## Figures and Tables

**Table 1:** Demographical features of subjects.

Feature	Value
Subject/number of eyes	28/42
Gender
Women	10
Men	18
Mean age	34.73 ± 6.50 (23–48)
Time of corneal ectasia diagnosis (months)	36.0 ± 16.92 (12–60)

**Table 2:** Preoperative clinical features of the subjects.

	Mean (min–max)
UDVA*	0.18 ± 0.22 (0.01–0.9)
CDVA**	0.46 ± 0.26 (0.01–1.0)
Spherical value (D)	−3.40 ± 4.29 (−18.0–0)
Cylindrical value (D)	−3.10 ± 1.59 (−7.5–0.75)
CTT (*μ*m)***	447.43 ± 42.41 (350–527)
*k*_1_ (D)****	45.10 ± 4.63 (32–57)
*k*_2_ (D)****	45.51 ± 5.64 (36–59)
*K*_mean._ (D)****	45.69 ± 4.63 (37–56)

*UDVA: uncorrected distance visual acuity, **CDVA: corrected distance visual acuity, ***CCT: central corneal thickness, and ****k_1_, *k*_2_, *k*_ort._: corneal curvature radii.

**Table 3:** Risk factors.

	*n*	Percent (%)
No risk	9	21.4
Risk present	33	78.6
FFK^1^	6	14.3
DA^2^	10	23.8
PMD^3^	2	4.8
Thin cornea	2	4.8
Steep cornea	3	7.1
Thin + steep cornea	2	4.8
DA + thin cornea	5	11.9
FFK + steep cornea	2	4.8
FFK + DA + steep cornea	1	2.4

^1^FFK: forme fruste keratoconus, ^2^DA: deep ablation, and ^3^PMD: pellucid marginal degeneration.
